# Interplay of microRNA and epigenetic regulation in the human regulatory network

**DOI:** 10.3389/fgene.2014.00345

**Published:** 2014-10-06

**Authors:** Matteo Osella, Andrea Riba, Alessandro Testori, Davide Corà, Michele Caselle

**Affiliations:** ^1^Dipartimento di Fisica, Istituto Nazionale di Fisica Nucleare, Università di TorinoTorino, Italy; ^2^Dipartimento di Oncologia, Istituto per la Ricerca sul Cancro di Candiolo-Istituto di Ricovero e Cura a Carattere Scientifico, Università di TorinoTorino, Italy

**Keywords:** microRNAs, epigenetic regulation, network motifs, feedback loops

## Abstract

The expression of protein-coding genes is controlled by a complex network of regulatory interactions. It is becoming increasingly appreciated that post-transcriptional repression by microRNAs, a class of small non-coding RNAs, is a key layer of regulation in several biological processes. In this contribution, we discuss the interplay between microRNAs and epigenetic regulators. Among the mixed genetic circuits composed by these two different kinds of regulation, it seems that a central role is played by double-negative feedback loops in which a microRNA inhibits an epigenetic regulator and in turn is controlled at the epigenetic level by the same regulator. We discuss a few relevant properties of this class of network motifs and their potential role in cell differentiation. In particular, using mathematical modeling we show how this particular circuit can exhibit a switch-like behavior between two alternative steady states, while being robust to stochastic transitions between these two states, a feature presumably required for circuits involved in cell fate decision. Finally, we present a list of putative double-negative feedback loops from a literature survey combined with bioinformatic analysis, and discuss in detail a few examples.

## 1. Introduction

MicroRNAs (miRNAs) are endogenous non-coding RNAs that negatively regulate the protein production of their mRNA targets in metazoans and plants. They are rather small (about 22 nucleotides long), single stranded RNAs, and are known to target a substantial portion of the human genome (Lewis et al., 2005; Friedman et al., 2009). They have been shown to play key roles in several biological processes ranging from development and metabolism to apoptosis and signaling pathways (Ambros, 2004; Bartel, 2004). Moreover their profiles are altered in several human diseases and in particular in cancer (Alvarez-Garcia and Miska, 2005; Esquela-Kerscher and Slack, 2006), making miRNAs a major focus of research in molecular biology.

**Figure 1:**
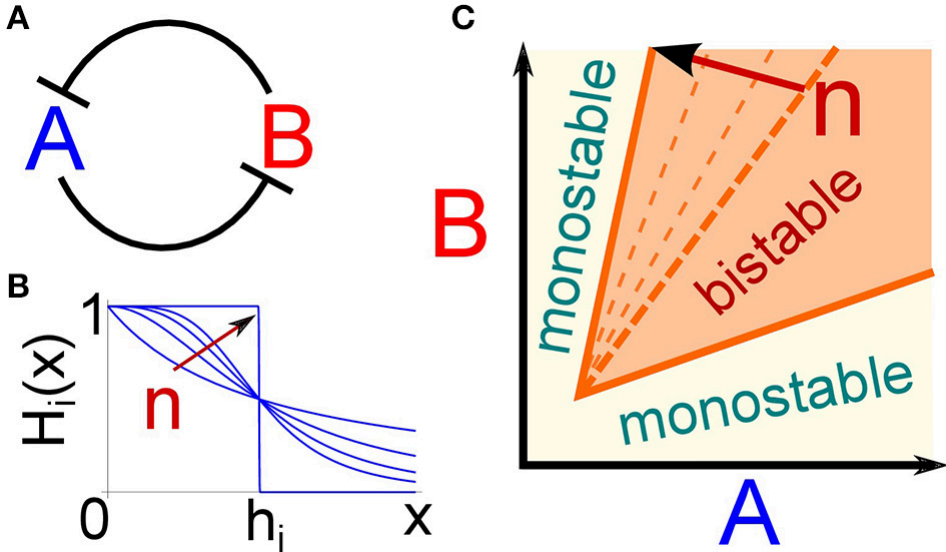
**The bistability region of the toggle switch depends on the degree of repression non-linearity. (A)** Cartoon of the interaction scheme that composes the toggle switch: the two genes A and B mutually repress each other. **(B)** The Hill function *H*_*i*_(*x*) describes how the production rate of gene *i* depends on the repressor concentration *x*. The exponent *n* defines the steepness of the curve. In the limit case of *n* → ∞, the Hill function becomes a step function with a critical repressor concentration *h*_*i*_ at which the target production is switched on. As we argue in the text, this limit case can be considered the suitable description for the transcriptional repression induced by epigenetic regulators such as chromating remodeling factors. **(C)** The bistability region of the toggle switch is depicted as a function of the amounts of the two gene products A and B. Increasing the steepness of the repressive function (i.e., the parameter *n*) enlarges the bistability region, thus extending the parameter range in which the circuit is suitable to implement cell fate decisions.

Recent work (see for instance Iorio et al., 2010; Kunej et al., 2011; Sato et al., 2011; Gruber and Zavolan, 2013; Wang et al., 2013) has shown that there is a strong interplay between miRNAs and epigenetic regulators. Their expression and mutual interactions are often highly coordinated, suggesting that epigenetic regulation can be fully understood by only considering epigenetic regulators and miRNAs as partners of a combined regulatory network. This combined network will be referred to as Epi-miRNA network in the following. Similarly to what happens in the purely transcriptional regulatory network (Alon, 2006, 2007), also in this Epi-miRNA network a few recurrent wiring patterns can be detected. These patterns are usually called network motifs. Among these small genetic circuits a special role seems to be played by the double negative feed-back loop (DNFL) in which a miRNA (or, in some cases, a set of miRNAs acting in a cooperative way) targets an epigenetic regulator, which in turn controls the expression of the same miRNA(s) (Figure 1A).

This network motif, which is usually called “toggle switch,” has several crucial functions. As suggested by the name, it may act as a genetic switch between different cell fates (Gardner et al., 2000), and as such it is found in several differentiation and developmental processes (Alon, 2006). At the same time it can be used as a “memory unit.” It is able to fix a transient stimulus into a stable expression pattern which persists even when the original stimulus disappears. In order to perform these functions in an optimal way the DNFL must be tuned so as to be in the bistability region (i.e., it must allow two competing stationary states), it must have a fine tuned switching threshold so as to avoid unwanted random transitions between the two alternative states (i.e., it must be a “robust” switch) but, at the same time, it should allow, if needed, a switch-back transition (i.e., in some cases it must be a “reversible” switch).

Even if there are several examples of toggle switches, and more generally of bistable circuits, in which both partners are transcription factors (Tian and Burrage, 2006), in the past few years there has been an increasing amount of evidence suggesting that in several biologically relevant realizations of the switch one of the two partners is a miRNA instead of a transcription factor. The typical example is the mir200-ZEB toggle switch, which is at the basis of the transitions between epithelial and mesenchymal phenotypes, and thus plays a crucial role in embryonic development as well as in cancer metastasis formation (Burk et al., 2008; Lu et al., 2013). In the following, we propose a possible advantage of miRNA regulation in this kind of cell-fate decision circuit. In particular, we show that miRNAs are better suited than transcription factors in conferring robustness to the committed cell decision. In fact, the physical mechanism of miRNA regulation can naturally increase the stability of the circuit steady states with respect to random transitions due to gene expression noise. This feature could be particularly relevant if each steady state is actually associated to a specific phenotype that has to be maintained in absence of specific stimuli, as is the case for the mir200-ZEB toggle switch. An intuitive way to understand it is to notice that miRNAs, acting at the post-transcriptional level, are in general able to decrease random fluctuations of target proteins (Levine et al., 2007; Osella et al., 2011), and this increases the robustness of the switch.

Moreover, we argue that this tendency is even stronger when one of the two partners of the toggle switch is an epigenetic regulator. Indeed, epigenetic regulation can be represented as a switch between discrete states of transcription of the target genes. Therefore, the response to variations in the concentration of epigenetic regulators is effectively highly non-linear and step-like, compared with the generally more graded dependence of target activity on its transcription factor concentrations. This feature, if combined with the post-trascriptional nature of miRNA regulation, increases the range of bistability of the switch and its overall robustness when it is inserted in a complex network with a wide range of possible inputs. This paper addresses these two issues by suitably modeling the toggle switch involving a miRNA and an epigenetic regulator. Finally, a list of possible candidate DNFLs will be provided and few relevant examples will be discussed in detail.

## 2. Results

### 2.1. Modeling the double negative feed-back loop: the role of non-linearity and epigenetic regulation

This section introduces the modeling framework used to represent the dynamics of the toggle switch, reviews the necessary conditions for the circuit to show a bistable behavior, and finally addresses the possible consequences of the presence of an epigenetic regulator as one node of a DNFL. The toggle switch is a network composed by two genes mutually repressing each other (Figure 1A). Since it is one of the simplest genetic circuits that can give rise to multiple equilibrium states, and thus in principle to cell fate decisions, it has been extensively studied (see for example Alon, 2006 and references therein). A coarse-grained mathematical description of the circuit dynamics can be expressed by the following couple of differential equations (Schultz et al., 2008):

$$\begin{array}{l} \frac{dA}{dt}=k_{A}H_{A}(B)-\gamma_{A}A\\ \frac{dB}{dt}=k_{B}H_{B}(A)-\gamma_{B}B \end{array}$$

in which only production and degradation of the two gene products A and B are considered, condensing in these two effective reactions several intermediate steps such as transcription, translation, mRNA export or maturation. Theoretical and empirical arguments suggest that repression can be modeled by making the production rate of each gene a non-linear function of the regulator concentration (Bintu et al., 2005a,b). More specifically, the production rate *k*_*i*_ of gene *i* is multiplied by a Hill function, shown in Figure 1B, of the regulator amount *x*:

$$H_{i}(x)=\frac{1}{1+{\left(\frac{x}{h_{i}}\right)}^{n_{i}}}.$$

The exponent *n* represents the cooperativity of the regulation and sets the steepness of the Hill function, while the effective dissociation constant *h* defines the regulator amount at which the production rate is half of its maximum value (Figure 1B).

Several mechanisms can increase the value of the exponent in the Hill function in Equation 2, making it sigmoidal-like, and thus inducing an ultrasensitive target response to changes in the repressor concentration (Zhang et al., 2013). In particular, epigenetic regulators, such as chromatin remodeling factors, are more likely to induce transitions between discrete states rather than smoothly change the transcription rate of genes in the targeted DNA regions. For example, histone modifications are usually modeled as transitions between a small number of chemical states with different DNA accessibility or affinity for the transcriptional machinery (Dodd et al., 2007), as it has indeed been shown in the particular case of DNA methylation (Lim and van Oudenaarden, 2007). Therefore, a specific epigenetic regulator will presumably make the target gene switch between two alternative transcription rates, corresponding to different chromatin states. This can be included in the proposed modeling scheme using a Hill function with an extremely high exponent *n*, thus effectively approaching a step function between two values of transcription probability.

Deterministic mathematical analysis allows to identify the conditions necessary to have bistability, which lets the system “decide” between two alternative expression states and ultimately between different phenotypes. Figure 1C shows the bistability region as a function of the concentrations of the two gene products (*A* and *B*). If the production rate of one of the two genes, and thus its concentration, is too high relative to the other, the circuit presents a unique possible steady state, otherwise it can relax to two different equilibria. These two alternative steady states correspond to one gene highly expressed while the other is switched off. Moreover, to ensure the presence of bistability, repression must be strong enough to avoid simultaneous expression of both genes, and a certain degree of non-linearity in the repression function is required (Schultz et al., 2008). In fact, if *n*_*i*_ = 1 in the Hill function (Equation 2) of both genes, the bistable region in Figure 1C vanishes. As discussed above, repression due to an epigenetic regulator, such as a chromatin remodeling factor, can be modeled using a high Hill exponent *n*, ensuring a certain degree of non-linearity in gene interaction. Figure 1C shows how increasing the value of *n* of one of the two regulatory functions leads to a progressively larger region of bistability. Therefore, the presence of an epigenetic regulator can naturally widen the parameter space in which the toggle switch presents two steady states, conferring robustness to the bistable behavior of the circuit.

### 2.2. Regulation by microRNAs increases the stability of the switch

Gene expression is inherently a stochastic process (Raj and van Oudenaarden, 2008). Fluctuations in protein concentration can induce random transitions between the alternative steady states of a bistable genetic circuit like the toggle switch in Figure 1A (Schultz et al., 2008). A bistable circuit at the basis of cell fate determination is expected to be robust to these stochastic transitions, since they could in principle drive the cell to an undesired phenotype. This section indeed focuses on the specific role that the nature of miRNA regulation can have in controlling the stochastic transitions between the two alternative steady states of a toggle switch.

A major source of stochasticity in gene expression is due to the burstiness in protein production. In fact, proteins have been observed to be produced in brief periods of high expression intensity followed by waiting periods (Friedman et al., 2006; Yu et al., 2006). This is mainly due to the fact that during the lifetime of a single mRNA several proteins can be produced, although also bursts of transcription, probably due to transitions in the promoter state, have been observed (Raj and van Oudenaarden, 2008). Therefore, a fluctuation at the transcriptional level can be amplified by the translation of a large protein burst stemming from a single mRNA. The average size of these bursts *b* is given by the product of the rate of translation *k*_*p*_ and the average lifetime of mRNA 1/γ_*m*_ (i.e., *b* = *k*_*p*_/γ_*m*_), while their frequency *a* is defined by the transcription rate *k*_*m*_ with respect to the timescale set by protein degradation γ_*p*_ (i.e., *a* = *k*_*m*_/γ_*p*_) (Friedman et al., 2006).

As a consequence, to fully account for the stochasticity in gene expression, a realistic mathematical description must take explicitly into account the transcription and translation processes, which combine to give rise to the observed bursts in protein production. Noise at the protein level can be evaluated analytically (Thattai and van Oudenaarden, 2001). In particular, it can be quantified measuring the relative fluctuations with the coefficient of variation $CV_{p}=\frac{\sigma_{p}}{\langle p\rangle }$ (where *p* represents the protein level), which for a constitutive gene takes the simple form

$$CV_{p}≃\frac{1+b}{\langle p\rangle },$$

where the mean protein level is simply given by the product of the average size of bursts and their frequency: 〈*p*〉 = *b a* (Thattai and van Oudenaarden, 2001; Friedman et al., 2006).

Regulation at the transcriptional level, like regulation by transcription factors, modulates the transcription rate of the target genes, thus affecting the burst frequency *a*. On the other hand, miRNAs are known to exert their action by suppressing translation (i.e., decreasing *k*_*p*_) or promoting mRNA degradation (increasing γ_*m*_) (Valencia-Sanchez et al., 2006). Both regulative modalities affect the target burst size rather than the frequency. Therefore, the same degree of repression exerted transcriptionally or post-transcriptionally via miRNAs will lead to very different levels of noise of the target protein concentration. In particular, the expression for the coefficient of variation in Equation 3 suggests that miRNAs, reducing the target burst size, are more effective in keeping fluctuations in gene expression under control. In fact, several circuits involving miRNA regulation have been suggested to play a role in conferring robustness to biological processes (Levine and Hwa, 2008; El Baroudi et al., 2011; Osella et al., 2011; Bosia et al., 2012; Ebert and Sharp, 2012; Riba et al., 2014).

These simple arguments indicate a possible evolutionary reason to prefer miRNA regulation, with respect to transcriptional regulation, to build toggle switches involved in cell fate decision. In fact, in this biological context a certain degree of robustness to stochastic fluctuations is probably a crucial feature. To further support this idea at a quantitative level, we introduce a more realistic and detailed mathematical description of toggle switches in the epi-miRNA network. The two steps of transcription and translation will be considered explicitely for the epigenetic regulator, in order to fully consider the stochastic effects arising from the burstiness of gene expression. Moreover, the physical coupling of miRNAs and target mRNAs, and the catalytic/stoichiometric nature of this coupling, will be taken into account. In fact, miRNA regulation is an example of molecular titration since it requires the direct one-to-one binding of a regulator and its target molecule (Buchler and Louis, 2008). The regulatory mechanism is thus different from transcriptional regulation, where even a small amount of transcription factors can influence the target production with no significant consequences on their concentration. Indeed, a mathematical description specifically designed for miRNA regulation has been previously introduced and some of its predictions tested experimentally (Levine et al., 2007; Levine and Hwa, 2008; Mukherji et al., 2011). This modeling strategy can be straightforwardly applied to the toggle switch here in analysis. Denoting with *s*, *m* and *p* the number of miRNAs, mRNAs and proteins respectively, the corresponding deterministic model of the circuit, describing the dynamics of the average amounts of the different molecules, is given by the following three coupled equations.

$$\begin{array}{l} \frac{ds}{dt}=\frac{k_{s}}{1+{\left(\frac{p}{h}\right)}^{n}}-\gamma_{s}s-\alpha kms\\ \frac{dm}{dt}=k_{m}-\gamma_{m}m-kms\\ \frac{dp}{dt}=k_{p}m-\gamma_{p}p. \end{array}$$

The parameter *k* represents the rate of miRNA-mRNA coupling (and will be dependent on the energy of RNA-RNA binding), while α is the cataliticity parameter describing the probability that a degradation event of a mRNA, induced by a miRNA, is accompained by the degradation of the miRNA itself. The limit α → 1 corresponds to a stoichiometric mode of action (as it is often the case for sRNA regulation in bacteria Levine et al., 2007), while the opposite situation of α → 0 represents a perfectly catalytic mode, in which the rate of mRNA degradation becomes simply a linear function of the number of miRNAs.

**Figure 2:**
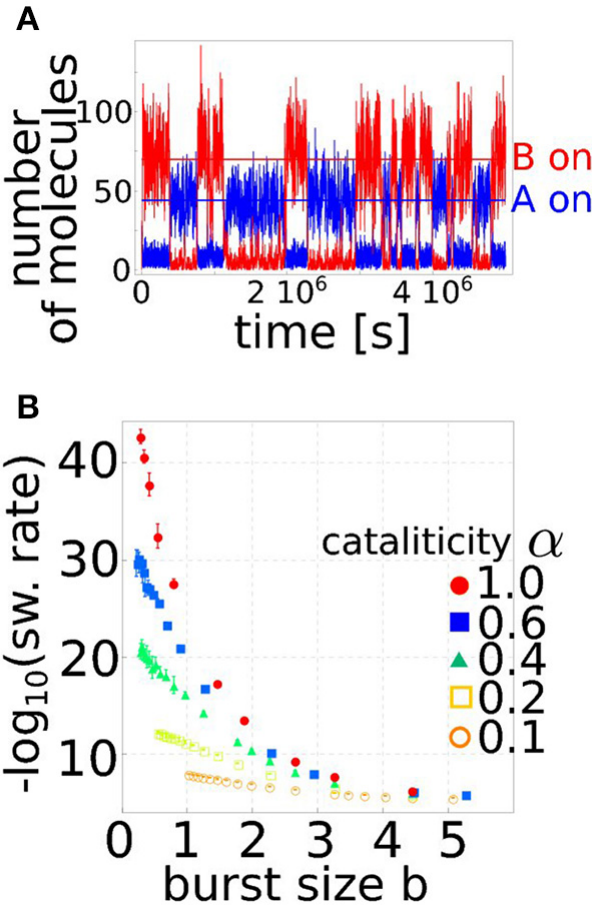
**Regulation by miRNAs increases the stability of the toggle switch by controlling the burst size. (A)** Stochastic fluctuations in gene expression can induce transitions between the two stable steady states. The figure shows an example of a simulation in which the circuit switches between the situation in which A is actively transcribed while B is switched off to the opposite one. The typical time between these transitions is set by the switching rate, which is a function of the circuit parameters. **(B)** The switching rate is shown as a function of the effective burst size $\left(b=\frac{k_{p}}{\gamma_{m}+k\;s}\right)$, as set by the level of miRNA regulation. The burst size is a major determinant of gene expression noise, and small variations in this parameter can vary the toggle switch stability of several order of magnitude. The different curves correspond to different levels of cataliticity α, i.e., the ability of the miRNA to be recycled and not degraded with the targeted mRNA (see Equation 4). The higher is the recycling ability (α → 0) the less is the circuit stability dependent on the burstiness of the process.

In order to evaluate the robustness of the circuit to stochastic transitions between the two steady states, the stochastic version of the model in Equation 4 has to be considered. In particular, the level of stability of a steady state is given by the typical time the system manages to dwell in it before a stochastic transition, and this time can be evaluated using stochastic simulations. Figure 2A shows an example of such simulations. The circuit randomly switches between the equilibrium in which gene A is on while B is shut off and the opposite state. The timing between these transitions defines the switching rate between the stable states. However, since the switching dynamics are often difficult to study directly with conventional computer simulations, because of the infrequent nature of the transitions, we used the “forward flux sampling” technique (Allen et al., 2005) to accumulate statistics on the switching rates over many realizations. Figure 2B summarizes the results of this analysis. As discussed above, miRNA regulation can control gene expression noise by reducing the target burst size. In fact, the effective burst size of protein *p* in the circuit can be defined as $b=\frac{k_{p}}{\gamma_{m}+ks}$ (as can be easily derived from Equation 4). This is indeed the actual mean number of proteins that can be produced from a mRNA, depending on the average miRNA concentration *s* and the strength of repression *k*. These two parameters are crucial in determining the amount of noise in the protein level, and thus the probability of observing a stochastic transition between the two steady states. In fact, the switching rate can change by several orders of magnitude depending on the level of miRNA regulation, and thus on the effective target burst size (Figure 2B). It should be stressed that a toggle switch composed only by transcriptional regulators would not be able to reduce the burst size of either of the two genes, and thus could not show the significative reduction in the switching rate that is on the other hand present for a miRNA-mediated toggle switch. Finally, the degree of cataliticity of the miRNA-mRNA interaction seems to play an important role in defining the stability of the circuit. A low degree of cataliticity, i.e., a high probability of a coupled miRNA-mRNA degradation after their physical interaction, allows a stronger reduction of the switching rate.

It is important to stress that (as we mentioned above in the case of the purely transcriptional toggle switch) Equation 4 represents a coarse-grained description of the actual dynamics of the regulatory circuit. At this level of resolution, several processes are condensed into few effective reactions. In particular, the processes involved in miRNA maturation and miRNA loading into the RISC (RNA-induced silencing complex) are neglected, and the availability of active miRNAs is just defined by the two parameters *k*_*s*_ and γ_*s*_. These processing steps are often intertwined in a non-trivial way. For instance, it has been recently shown (Winter and Diederichs, 2011; Martinez and Gregory, 2013) that the stability of the miRNA is strongly influenced by its binding to the RISC. When the concentration of proteins crucial for the RISC formation, such as Argonaute proteins, is high enough or well coupled with the miRNA concentration, as suggested by recent experiments (Martinez and Gregory, 2013), the coarse-grained description in Equation 4 is effective, with the parameter γ_*s*_ capturing the stability of the active miRNA loaded into the RISC complex. However, in the presence of saturation of proteins needed for the RISC formation (or other necessary small RNA processing or transport machinery), as it could be the case in transfection experiments (Khan et al., 2009), more detailed models must be considered to fully capture the circuit dynamics.

Moreover, the dynamics of miRNA loading into the RISC can introduce delays between miRNA transcription and the regulatory effect on their targets (Hausser et al., 2013). These delays can be effectively included in computational models and can indeed quantitatively affect the circuit dynamics (Osella et al., 2011). However, miRNAs are still effective in the control of target fluctuations as long as these delays are not too large (Osella et al., 2011).

Therefore, the proposed coarse-grained model is sufficient to analyse and compare the robustness and stability against fluctuations of different types of toggle switches (which is the main goal of our work). However, it is clear that a more refined description of the regulatory circuit should keep into account also additional molecular players, and in particular the miRNA-RISC interaction, in order to be able to formulate precise quantitative predictions.

### 2.3. Bioinformatic search of candidate double negative feed-back loops in the epi-miRNA network

The identification with bioinformatic methods of regulatory motifs involving epigenetic regulators is much more difficult than the analogous search in the case of regulatory motifs composed by Transcription Factors or miRNAs. The reasons are the lack of precise sequence motifs and the strong dependence on tissue type and cell state of epigenetic regulation. Since it is not possible to rely on sequence information, the main tool to address the problem is ChIP-seq data for the main epigenetic markers. Keeping into account these features we decided to address the problem with a two step analysis.

**Figure 3:**
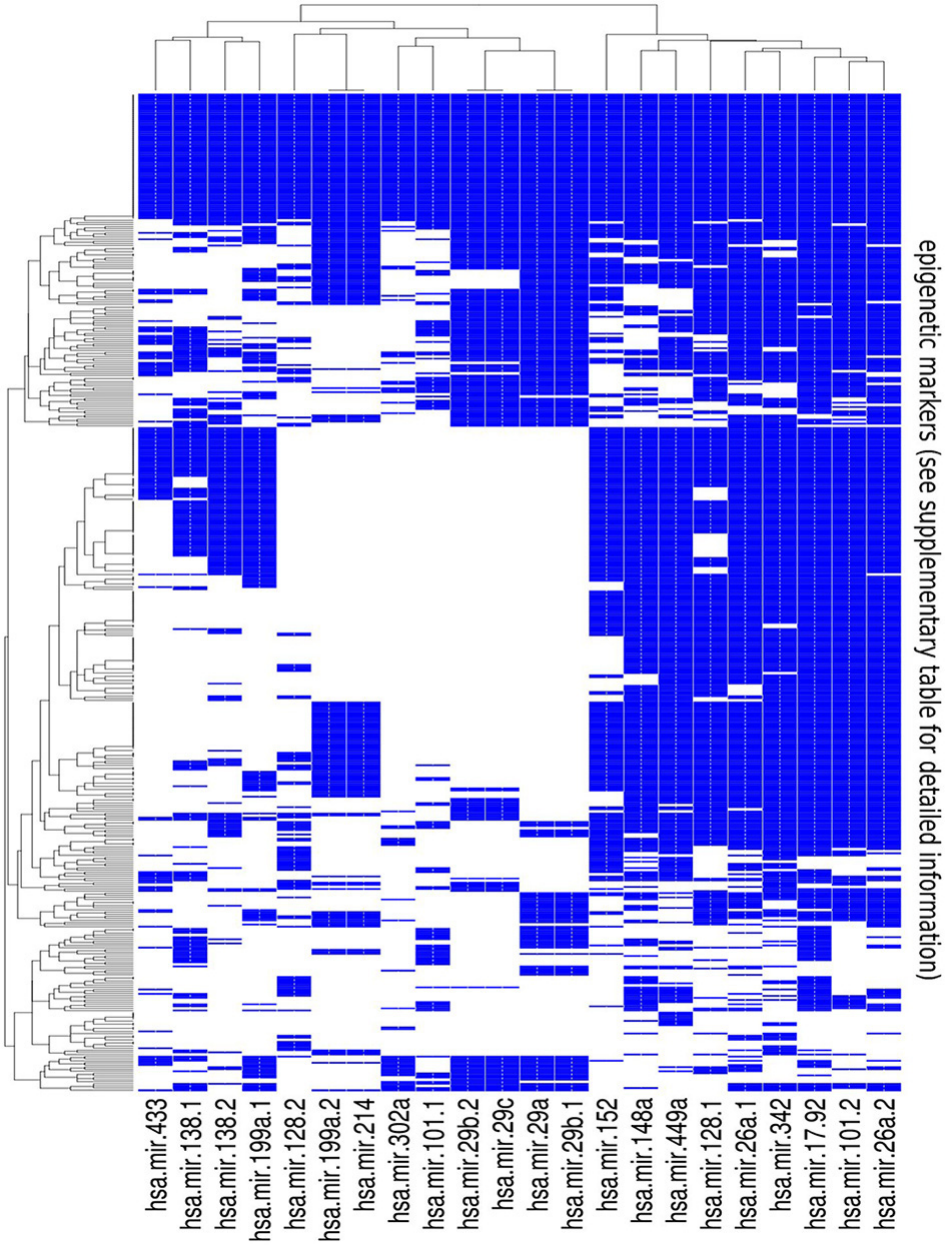
**Summary of the results of the bionformatic analysis.** The heatmap is a graphical representation of the matrix obtained by annotating all available histone modifications and methylation patterns from the ENCODE epigenetic tracks at the promoters of the pre-miRNAs listed in Table 1. Color code: blue whether overlap exists between the putative pre-miRNA promoter and the corresponding epigenetic track, and white otherwise. Detailed information on the epigenetic markers listed in the rows of the map can be found in the supplementary material.

- First we identified, using literature information (Iorio et al., 2010; Kunej et al., 2011; Sato et al., 2011; Gruber and Zavolan, 2013; Wang et al., 2013) and convergent signatures from existing databases a list of miRNAs which are known to target a few specific epigenetic regulators. We performed this analysis using the same tools we developed for our previous studies of motifs involving miRNAs (Re et al., 2009; Friard et al., 2010). The result of this first step is reported in Table 1.
- Second, we screened the ENCODE data for epigenetic markers looking for signatures in the promoter regions of the above miRNAs. We summarized the results of this screening into a matrix (included in the supplementary material) whose construction is discussed in the Material and Methods Section. The results are also reported as a heatmap in Figure 3.

**Table 1:** **List of miRNAs targeting epigenetic regulators**.

**miRNA**	**target**
has-miR302a	MECP2
hsa-miR29a	TET1, TET2, TET3
has-miR29a/c	DNMT3A, DNMT3B
has-miR29b-1/2	DNMT1 (Indirect via SP1)
hsa-miR148a	DNMT3B
hsa-miR148a	DNMT1
hsa-miR152	DNMT1
has-miR302a	DNMT1 (Indirect via AOF2)
hsa-miR342	DNMT1
hsa-miR17-92	DNMT1
hsa-miR26a-1/2	EZH2
hsa-miR101-1/2	EZH2/EED
hsa-miR214	EZH2
hsa-miR128-1/2	BMI-1
hsa-miR199a-1/2	BRM
hsa-miR433	HDAC6
hsa-miR449a	HDAC1
hsa-miR138	SIRT1

In the first column we report a list of miRNAs which are known to target epigenetic regulators and in the second column the corresponding targets. We use the notation hsa-miR17-92 to denote the whole cluster comprising hsa-miR17, hsa-miR18a, hsa-miR19a/b, hsa-miR20a, hsa-miR92a. We keep them together since they are part of an unique transcript and are controlled by a common promoter. Thus they will be associated to a single column in Figure 3.

This matrix allows to browse the epigenetic regulation of the selected miRNAs. As expected, it gives a rather fuzzy representation of it, with a strong tissue dependence. Nevertheless, it allows to identify a few precise patterns which are highlighted by the clustering structure reported in the heatmap and to select a few candidate closed feedback loops which are discussed in Section 4.2.

## 3. Materials and methods

### 3.1. Construction of the heatmap of Figure 3

The heatmap reported in Figure 3 is a graphical representation of the matrix obtained by annotating histone modifications and methylation patterns from the ENCODE epigenetic tracks at the promoters of the pre-miRNAs listed in Table 1. We retrieved the data from http://genome.ucsc.edu/ENCODE/downloads.html (Raney et al., 2011; Rosenbloom et al., 2013). We first downloaded DNA methylation profiles, obtained using Reduced Representation Bisulfite Sequencing (RRBS) or with the Infinium Human Methylation 450 platform, which uses bisulfite treated genomic DNA to assay the methylation status of more than 450,000 CpG sites covering all designatable RefSeq genes (all these data are from Hudson Alpha Institute of Biotechnology). We next downloaded histone modifications obtained by ChIP-sequencing, which include histone variant H2AZ, methylation status of the 4th, 36th, and 79th residue of histone 3, acetylation and methylation status of the 9th and 27th residue of histone 3 and monomethylation of the 20th residue from H4. All these experiments were performed on different cell lines and in some cases replicates are available.

We annotated all datasets at promoters from pre-miRNAs of interest (defined as 2000 nts upstream of the pre-miRNA TSS) and marked the entries of the matrix with 1 when there was at least 1 nt of overlap between the promoter and the chosen epigenetic track and with zero if no overlap was present.

In-house perl scripts were used to annotate epigenetic modifications in the promoters of selected pre-miRNAs. The heatmap in Figure 3 was generated with the R package “gplots” (function “heatmap.2”). The source code for this analysis is available at https://github.com/atestori/Interplay_of_microRNA_and_epigenetic_regulation_in_the_human_regulatory_network/releases/tag/code1.

## 4. Discussion

### 4.1. Analysis of bioinformatic results

It can be seen from the heatmap of Figure 3, and from the corresponding matrix in the supplementary material, that the epigenetic regulation of the selected miRNAs is characterized by an intricated and fuzzy network of interactions which are characterized by a few general features:

- Looking at the structure of the columns we see that it often happens that different epigenetic mechanisms act synergistically to regulate the same miRNA. This is in our opinion the most interesting outcome of this analysis and will be confirmed by the examples that we discuss in detail in Section 4.2.
- In several cases the miRNAs that we selected overlap with the epigenetic markers mediated by exactly the same genes which they target. These are, within the limits of our analysis, good candidates to realize the DNFL which we studied in the previous sections. We selected among them a few cases (discussed in Section 4.2).
- The miRNAs seem to be regulated in a coordinated way. Looking at the rows of the heatmap, it can be noticed that in several cases the same epigenetic marker is present in several miRNAs at the same time, thus realizing also on this side a combinatorial and synergistic layer of regulation.

The main lesson that we learn from this analysis is that the mixed epi-miRNA network is strongly interconnected and that when one of the toggle switches is activated, it is likely to activate also other switches, triggering a complex sequence of regulatory steps which all contribute to fix the final state of the system. This may be better understood by looking at a few concrete examples.

### 4.2. A few examples of double negative feed-back loops in the epi-miRNA network

This section considers a few examples of DNFLs. We discuss a few cases for which a whole experimental knowledge is available both of the inhibitory interactions and of the different cell fates associated to the two competing states of the switch. In particular, we chose examples that involve all the most important epigenetic mechanisms, also trying to underline the remarkable connections linking them together.

#### 4.2.1. The Ezh2 - mir214 loop

This was one of the first DNFL identified in the Epi-miRNA network (Juan et al., 2009) and is probably one of the best studied examples. Ezh2 is the catalytic subunit of the Polycomb complex and with Suz12 and Eed (which is also involved in a DNFL, see Section 4.2.2) is part of the Polycomb Repressive complex 2 (PRC2) which mediates Histone H3K27 trimethylation (H3K27me3). This complex plays a crucial role in gene silencing via chromatin condensation. Among the targets of PRC2 there are several miRNAs and in particular one of its targets is mir-214. In turn it was shown in Juan et al. (2009) that Ezh2 is targeted by mir-214 thus closing a DNFL. Mir-214 is located in one of the introns of the gene DNM3 on the opposite strand orientation, thus it is controlled by its own promoter. It is located at less than 5kb of distance from another important miRNA: mir199a which, remarkably enough, is involved in another epigenetic feedback loop (Section 4.2.3). Mir-214 is vertebrate-specific and is known to be involved in several types of cancer. In particular, it is thought to encourage the metastasis of melanoma and is known to be downregulated in human cervical cancer. The DNFL involving Ezh2 and mir-214 has been studied in detail in Juan et al. (2009) where it was shown its role in inducing the differentiation of embryonic stem cells into skeletal muscle cells. Before differentiation the Polycomb complex is upregulated and represses the transcription of a large set of genes among which also mir-214. Upon differentiation, stimulated by the MyoD and myogenin developmental regulators, mir-214 is released from the Polycomb control and downregulates the Ezh2 translation thus accelerating the differentiation of skeletal muscle cells.

#### 4.2.2. The Ezh2/Eed - mir101 loop

This DNFL is strictly related to the previous one. The most interesting feature is that this time the miRNA targets two of the three subunits of the PRC2 complex thus ensuring an even more effective downregulation of its activity. This loop was studied in detail in Wang et al. (2013) where its role in controlling hepatocarcinogenesis was shown. In particular, it was shown that when mir-101 is repressed and PRC2 upregulated the formation of malignant phenotypes of Hepatocellular Carcinoma cells is increased, leading to poorer prognosis in patients. When instead mir-101 is expressed and PRC2 repressed, malignant phenotypes are suppressed and the prognosis improves. It was also shown that the switch of this DNFL may be triggered by the oncogene c-Myc, which is able to mediate and increase the repression of mir-101 by PRC2.

#### 4.2.3. The BRM - mir199 loop

This DNFL is particularly interesting since it involves a different epigenetic mechanism: the SWI/SNF pathway. However, it is deeply linked with the previous ones, since mir-199 is located in the same cluster of mir214 and is known to form a common precursor with mir-214 (Loebel et al., 2005), and is thus controlled by the same PRC2 complex discussed in the previous examples. The SWI/SNF proteins form a chromatin remodeling complex (for an updated review on the SWI/SNF pathway see for instance Wilson and Roberts, 2011) which is known to interact in a lineage specific manner with other chromatin remodeling complexes and among them in particular with PRC2 (Ho et al., 2011). The complex may contain two catalytic subunits. One of them is the Brm gene (also known as Smarca2) whose translation is controlled by mir-199 (Sakurai et al., 2011). Interestingly, the 3′UTR region of Brm is targeted by both the mature versions of mir-199, i.e., mir-199-3p and mir-199-5p (Sakurai et al., 2011). This is a rather uncommon situation and is typically the signature of a strong post-transcriptional control of the miRNA on its target. In turn Brm is able to silence the mir-199, mir-214 cluster by silencing Egr1 which is known to be a strong activator of the cluster (Sakurai et al., 2011), thus closing in an indirect way a double negative feedback loop between Brm and mir-199. This toggle switch is known to play an important role in several types of cancer leading to different cell populations during oncogenesis, thus explaining why mir-199 had been reported as an ambiguous marker in several types of cancer, being either upregulated or downregulated in different samples of the same tumor.

#### 4.2.4. The DNMT1 - mir17-92 loop

Another crucial layer of epigenetic regulation is DNA methylation which is mediated by Dnmt proteins. It is well known (see for instance Gruber and Zavolan, 2013) that Dnmt proteins are strictly controlled in a coordinated way by a number of miRNAs, among them in particular mir-29a/b/c, mir-152, mir148a, mir342, mir302 and various members of the cluster mir17-92. This last cluster has recently attracted particular attention since it has been shown that the cluster itself is controlled at the level of DNA methylation by the same Dnmt1 protein which is targeted by the miRNA of the cluster thus closing again a DNFL (Dakhlallah et al., 2013). This loop has been shown to play an important role in controlling the pathogenesis of lung fibrosis (Dakhlallah et al., 2013). In particular it has been shown that in patients affected by Idiopathic Pulmonary Fibrosis the miRNAs of the cluster are downregulated and Dnmt1 is upregulated with respect to control samples (Dakhlallah et al., 2013), thus suggesting that the disease could be associated to a switch of this DNFL.

#### 4.2.5. The Sirt1 - mir138 loop

Sirt1 is a NAD dependent histone deacetylase, it is part of a large family of histone deacetylase proteins that represent yet another layer of epigenetic regulation. It acts as a transcriptional repressor by inducing a compact chromatin structure. It has been recently shown in Liu et al. (2013) that Sirt1 is controlled by mir-138 which in turn is repressed by Sirt1. This DNFL also triggers mammalian axon regeneration. In particular it was shown in Liu et al. (2013) that mir-138 is a suppressor of axon regeneration and that the switch between mir138 and Sirt1 is able to regulate mammalian axon regeneration *in vivo*. In the same paper it was also described that such a switch occurs in response to peripheral nerve injury.

## 5. Conclusions

**Figure 4:**
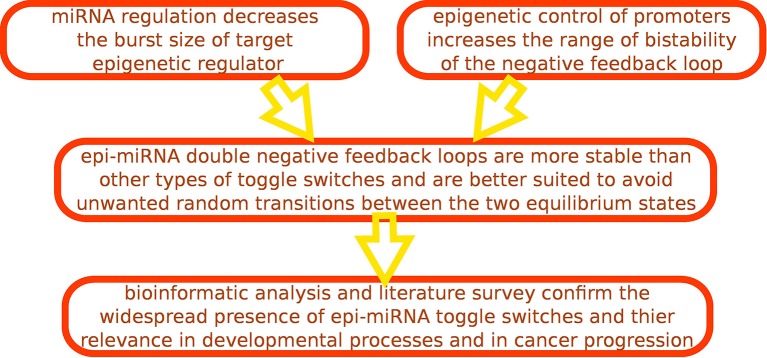
**Workflow of our analysis**.

As we have seen, in the human regulatory network there is a strong interplay between miRNAs and epigenetic regulators. Apparently, there was a strong evolutionary pressure to develop regulatory circuits combining these two types of regulations in the context of cell-decision making, and in particular several “epi-miRNA” toggle switches can be identified. One of the aims of our work was precisely to suggest possible reasons behind this evolutionary pressure. To this end, we studied both the deterministic and the stochastic behavior of this switch, and compared it with other possible choices, in which, instead of a miRNA or an epigenetic regulator, a transcription factor is the regulator. Our main result is that the epi-miRNA combination is the one which ensures the widest range of bistability and robustness of the two equilibrium states of the switch, as summarized in the sketch in Figure 4. This makes this type of circuit perfectly suited for robust but reversible decisions and indeed these circuits are typically involved in cell fate determination and development. First of all, epigenetic regulators playing a role in development are usually global regulators (Chen and Dent, 2014), affecting the expression of several genes. Therefore, the decision made at the level of expression of these master regulators can be propagated to a large downstream expression program, potentially defining the cellular phenotype. This feature suggests a possible reason for the presence of epigenetic regulation in cell decision making circuits such as toggle switches. Moreover, as Figure 1C indicates, the binary-like nature of epigenetic repression naturally increases the robustness of the bistable behavior of the circuit, thus ensuring the competition between alternative steady states for a large set of parameter values.

On the other side, we have shown that the very nature of post-transcriptional regulation by miRNAs can keep under control undesired random transitions between the two possible equilibrium states by reducing the target burst size. The combination of these features suggests an evolutionary reason for the specific interplay between epigenetic regulators and miRNAs in bistable circuits that have to implement stable and long-lasting cell fate decisions.

Interestingly, the combination of the two characteristics discussed above leads to a controlled reversibility of the toggle switch. In a generic toggle switch a transition occurs when a strong enough external stimulus acts on one of the two players of the switch. The only condition is that the stimulus must be larger than the “potential barrier” between the two equilibrium states. A well studied example is, for instance, the epithelial-mesenchymal transition controlled by the mir200-ZEB circuit (Lu et al., 2013). However, in the “epi-miRNA” case we have a further level of complexity since the potential barrier depends on the epigenetic control on the miRNA promoter, and this epigenetic control is particularly sensitive to the cellular environment (Feil and Fraga, 2011). The dependence of epigenetic modifications on environmental factors can thus change the stability of the switch, and eventually drive switch-back transitions due to sudden modifications of the potential barrier. Thus, the epi-miRNA toggle switch can ensure a strong stability to random transitions in homeostatic situations, but has a certain level of plasticity that can be crucial to induce a phenotype switch in the presence of changes in the cellular environment, for example due to transitions between different developmental stages (Feil and Fraga, 2011). It would be very interesting to include also this feature in our model. However, a detailed model of the epigenetic interaction and its relations with the cellular environment would be required, and this is still an open issue (Dodd et al., 2007).

In conclusion, the analysis presented in this paper suggests a possible relevant role for epi-miRNA DNFLs in cellular decision making. The peculiar nature of epigenetic and miRNA regulations can ensure a bistable circuit behavior in a vast range of conditions while keeping the alternative steady states robust with respect to stochastic fluctuations.

## Author contributions

Matteo Osella and Michele Caselle designed the research; Matteo Osella, Andrea Riba, Alessandro Testori, Davide Corà, Michele Caselle performed the research; Andrea Riba, Alessandro Testori, Davide Corà analyzed the data; Matteo Osella and Michele Caselle wrote the paper. All the authors read and approved the final manuscript.

## Funding

This work was partially funded by the FSP grant GeneRNet.

### Conflict of interest statement

The authors declare that the research was conducted in the absence of any commercial or financial relationships that could be construed as a potential conflict of interest.

## Supplementary material

We include as Supplementary Material a matrix with the data used to construct the heatmap of Figure 3. Each column corresponds to one of the chosen miRNAs while the rows correspond to different epigenetic markers. To allow the identification of the different markers we used the following convention: we added to the names of the downloaded files a prefix which refers to the cell line used in the experiment. If the dataset does not refer to methylation profiles, we also added at the very beginning the antibody used in the ChIP-seq experiment (these features are separated by an underscore). Methylation datasets therefore start with the name of a cell line.

## References

[B1] Allen R. J. Warren P. B. Ten Wolde P. R. (2005). Sampling rare switching events in biochemical networks. Phys. Rev. Lett. 94:018104 10.1103/PhysRevLett.94.018104 15698138

[B2] Alon U. (2006). An Introduction to Systems Biology: Design Principles of Biological Circuits. Boca Raton, FL: Chapman and Hall/ CRC Press

[B3] Alon U. (2007). Network motifs: theory and experimental approaches. Nat. Rev. Genet. 8, 450–461 10.1038/nrg2102 17510665

[B4] Alvarez-Garcia I. Miska E. A. (2005). MicroRNA functions in animal development and human disease. Development 132, 4653–4662 10.1242/dev.02073 16224045

[B5] Ambros V. (2004). The functions of animal microRNAs. Nature 431, 350–355 10.1038/nature02871 15372042

[B6] Bartel D. P. (2004). MicroRNAs: genomics, biogenesis, mechanism, and function. Cell 116, 281–297 10.1016/S0092-8674(04)00045-5 14744438

[B7] Bintu L. Buchler N. E. Garcia H. G. Gerland U. Hwa T. Kondev J. (2005a). Transcriptional regulation by the numbers: applications. Curr. Opin. Genet. Dev. 15, 125–135 10.1016/j.gde.2005.02.006 15797195 PMC3462814

[B8] Bintu L. Buchler N. E. Garcia H. G. Gerland U. Hwa T. Kondev J. (2005b). Transcriptional regulation by the numbers: models. Curr. Opin. Genet. Dev. 15, 116–124 10.1016/j.gde.2005.02.007 15797194 PMC3482385

[B9] Bosia C. Osella M. Baroudi M. E. Corà D. Caselle M. (2012). Gene autoregulation via intronic microRNAs and its functions. BMC Syst. Biol. 6:131 10.1186/1752-0509-6-131 23050836 PMC3534558

[B10] Buchler N. E. Louis M. (2008). Molecular titration and ultrasensitivity in regulatory networks. J. Mol. Biol. 384, 1106–1119 10.1016/j.jmb.2008.09.079 18938177

[B11] Burk U. Schubert J. Wellner U. Schmalhofer O. Vincan E. Spaderna S. (2008). A reciprocal repression between zeb1 and members of the mir-200 family promotes emt and invasion in cancer cells. EMBO Rep. 9, 582–589 10.1038/embor.2008.74 18483486 PMC2396950

[B12] Chen T. Dent S. Y. R. (2014). Chromatin modifiers and remodellers: regulators of cellular differentiation. Nat. Rev. Genet. 15, 93–106 10.1038/nrg3607 24366184 PMC3999985

[B13] Dakhlallah D. Batte K. Wang Y. Cantemir-Stone C. Z. Yan P. Nuovo G. (2013). Epigenetic regulation of miR-17 similar to 92 contributes to the pathogenesis of pulmonary fibrosis. Am. J. Res. Crit. Care Med. 187, 397–405 10.1164/rccm.201205-0888OC 23306545 PMC3603596

[B14] Dodd I. B. Micheelsen M. A. Sneppen K. Thon G. (2007). Theoretical analysis of epigenetic cell memory by nucleosome modification. Cell 129, 813–822 10.1016/j.cell.2007.02.053 17512413

[B15] Ebert M. S. Sharp P. A. (2012). Roles for microRNAs in conferring robustness to biological processes. Cell 149, 515–524 10.1016/j.cell.2012.04.005 22541426 PMC3351105

[B16] El Baroudi M. Corà D. Bosia C. Osella M. Caselle M. (2011). A curated database of miRNA mediated feed-forward loops involving MYC as master regulator. PLoS ONE 6:e14742 10.1371/journal.pone.0014742 21390222 PMC3048388

[B17] Esquela-Kerscher A. Slack F. J. (2006). Oncomirs - microRNAs with a role in cancer. Nat. Rev. Cancer 6, 259–269 10.1038/nrc1840 16557279

[B18] Feil R. Fraga M. F. (2011). Epigenetics and the environment: emerging patterns and implications. Nat. Rev. Genet. 13, 97–109 10.1038/nrg3142 22215131

[B19] Friard O. Re A. Taverna D. De Bortoli M. Corà D. (2010). CircuitsDB: a database of mixed microRNA/transcription factor feed-forward regulatory circuits in human and mouse. BMC Bioinformatics 11:435 10.1186/1471-2105-11-435 20731828 PMC2936401

[B20] Friedman N. Cai L. Xie X. S. (2006). Linking stochastic dynamics to population distribution: an analytical framework of gene expression. Phys. Rev. Lett. 97:168302 10.1103/PhysRevLett.97.168302 17155441

[B21] Friedman R. C. Farh K. K.-H. Burge C. B. Bartel D. P. (2009). Most mammalian mRNAs are conserved targets of microRNAs. Genome Res. 19, 92–105 10.1101/gr.082701.108 18955434 PMC2612969

[B22] Gardner T. Cantor C. Collins J. (2000). Construction of a genetic toggle switch in *Escherichia coli*. Nature 403, 339–342 10.1038/35002131 10659857

[B23] Gruber A. J. Zavolan M. (2013). Modulation of epigenetic regulators and cell fate decisions by miRNAs. Epigenomics 5, 671–683 10.2217/epi.13.65 24283881

[B24] Hausser J. Syed A. P. Selevsek N. van Nimwegen E. Jaskiewicz L. Aebersold R. (2013). Timescales and bottlenecks in miRNA-dependent gene regulation. Mol. Syst. Biol. 9:711 10.1038/msb.2013.68 24301800 PMC3882800

[B25] Ho L. Miller E. L. Ronan J. L. Ho W. Q. Jothi R. Crabtree G. R. (2011). esBAF facilitates pluripotency by conditioning the genome for LIF/STAT3 signalling and by regulating polycomb function. Nat. Cell Biol. 13, 903–913 10.1038/ncb2285 21785422 PMC3155811

[B26] Iorio M. V. Piovan C. Croce C. M. (2010). Interplay between microRNAs and the epigenetic machinery: an intricate network. Biochim. Biophys. Acta Gene Regul. Mech. 1799, 694–701 10.1016/j.bbagrm.2010.05.005 20493980

[B27] Juan A. H. Kumar R. M. Marx J. G. Young R. A. Sartorelli V. (2009). Mir-214-dependent regulation of the polycomb protein Ezh2 in skeletal muscle and embryonic stem cells. Mol. Cell 36, 61–74 10.1016/j.molcel.2009.08.008 19818710 PMC2761245

[B28] Khan A. A. Betel D. Miller M. L. Sander C. Leslie C. S. Marks D. S. (2009). Transfection of small RNAs globally perturbs gene regulation by endogenous microRNAs. Nat. Biotechnol. 27, 549–555 10.1038/nbt0709-671a 19465925 PMC2782465

[B29] Kunej T. Godnic I. Ferdin J. Horvat S. Dovc P. Calin G. A. (2011). Epigenetic regulation of microRNAs in cancer: an integrated review of literature. Mutat. Res. Fund. Mol. Mech. Mutagene. 717, 77–84 10.1016/j.mrfmmm.2011.03.008 21420983

[B30] Levine E. Hwa T. (2008). Small RNAs establish gene expression thresholds. Curr. Opin. Microbiol. 11, 574–579 10.1016/j.mib.2008.09.016 18935980 PMC2613760

[B31] Levine E. Zhang Z. Kuhlman T. Hwa T. (2007). Quantitative characteristics of gene regulation by small RNA. PLoS Biol. 5:e229 10.1371/journal.pbio.0050229 17713988 PMC1994261

[B32] Lewis B. P. Burge C. B. Bartel D. P. (2005). Conserved seed pairing, often flanked by adenosines, indicates that thousands of human genes are microRNA targets. Cell 120, 15–20 10.1016/j.cell.2004.12.035 15652477

[B33] Lim H. N. van Oudenaarden A. (2007). A multistep epigenetic switch enables the stable inheritance of dna methylation states. Nat. Genet. 39, 269–275 10.1038/ng1956 17220888

[B34] Liu C.-M. Wang R.-Y. Saijilafu, Jiao Z.-X. Zhang B.-Y. Zhou F.-Q. (2013). MicroRNA-138 and SIRT1 form a mutual negative feedback loop to regulate mammalian axon regeneration. Genes Dev. 27, 1473–1483 10.1101/gad.209619.112 23796896 PMC3713428

[B35] Loebel D. Tsoi B. Wong N. Tam P. (2005). A conserved noncoding intronic transcript at the mouse Dnm3 locus. Genomics 85, 782–789 10.1016/j.ygeno.2005.02.001 15885504

[B36] Lu M. Jolly M. K. Levine H. Onuchic J. N. Ben-Jacob E. (2013). Microrna-based regulation of epithelial-hybrid-mesenchymal fate determination. Proc. Natl. Acad. Sci. U.S.A. 110, 18144–18149 10.1073/pnas.1318192110 24154725 PMC3831488

[B37] Martinez N. J. Gregory R. I. (2013). Argonaute2 expression is post-transcriptionally coupled to microRNA abundance. RNA Publi. RNA Soc. 19, 605–612 10.1261/rna.036434.112 23485552 PMC3677276

[B38] Mukherji S. Ebert M. S. Zheng G. X. Y. Tsang J. S. Sharp P. A. van Oudenaarden A. (2011). Micrornas can generate thresholds in target gene expression. Nat. Genet. 43, 854–859 10.1038/ng.905 21857679 PMC3163764

[B39] Osella M. Bosia C. Corà D. Caselle M. (2011). The role of incoherent microRNA-mediated feedforward loops in noise buffering. PLoS Comput. Biol. 7:e1001101 10.1371/journal.pcbi.1001101 21423718 PMC3053320

[B40] Raj A. van Oudenaarden A. (2008). Nature, nurture, or chance: stochastic gene expression and its consequences. Cell 135, 216–226 10.1016/j.cell.2008.09.050 18957198 PMC3118044

[B41] Raney B. J. Cline M. S. Rosenbloom K. R. Dreszer T. R. Learned K. Barber G. P. (2011). ENCODE whole-genome data in the UCSC genome browser (2011 update). Nucleic Acids Res. 39, D871–D875 10.1093/nar/gkq1017 21037257 PMC3013645

[B42] Re A. Corà D. Taverna D. Caselle M. (2009). Genome-wide survey of microRNA-transcription factor feed-forward regulatory circuits in human. Mol. Biosyst. 5, 854–867 10.1039/b900177h 19603121 PMC2898627

[B43] Riba A. Bosia C. Baroudi M. E. Ollino L. Caselle M. (2014). A combination of transcriptional and microrna regulation improves the stability of the relative concentrations of target genes. PLoS Comput. Biol. 10:e1003490 10.1371/journal.pcbi.1003490 24586138 PMC3937125

[B44] Rosenbloom K. R. Sloan C. A. Malladi V. S. Dreszer T. R. Learned K. Kirkup V. M. (2013). ENCODE data in the UCSC genome browser: year 5 update. Nucleic Acids Res. 41, D56–D63 10.1093/nar/gks1172 23193274 PMC3531152

[B45] Sakurai K. Furukawa C. Haraguchi T. Inada K.-I. Shiogama K. Tagawa T. (2011). MicroRNAs miR-199a-5p and-3p target the Brm Subunit of SWI/SNF to generate a double-negative feedback loop in a variety of human cancers. Cancer Res. 71, 1680–1689 10.1158/0008-5472.CAN-10-2345 21189327

[B46] Sato F. Tsuchiya S. Meltzer S. J. Shimizu K. (2011). MicroRNAs and epigenetics. FEBS J. 278, 1598–1609 10.1111/j.1742-4658.2011.08089.x 21395977

[B47] Schultz D. Walczak A. M. Onuchic J. N. Wolynes P. G. (2008). Extinction and resurrection in gene networks. Proc. Natl. Acad. Sci. U.S.A. 105, 19165–19170 10.1073/pnas.0810366105 19033463 PMC2614733

[B48] Thattai M. van Oudenaarden A. (2001). Intrinsic noise in gene regulatory networks. Proc. Natl. Acad. Sci. U.S.A. 98, 8614–8619 10.1073/pnas.151588598 11438714 PMC37484

[B49] Tian T. Burrage K. (2006). Stochastic models for regulatory networks of the genetic toggle switch. Proc. Natl. Acad. Sci. U.S.A. 103, 8372–8377 10.1073/pnas.0507818103 16714385 PMC1482501

[B50] Valencia-Sanchez M. A. Liu J. Hannon G. J. Parker R. (2006). Control of translation and mrna degradation by mirnas and sirnas. Genes Dev. 20, 515–524 10.1101/gad.1399806 16510870

[B51] Wang Z. Yao H. Lin S. Zhu X. Shen Z. Lu G. (2013). Transcriptional and epigenetic regulation of human microRNAs. Cancer Lett. 331, 1–10 10.1016/j.canlet.2012.12.006 23246373

[B52] Wilson B. G. Roberts C. W. M. (2011). SWI/SNF nucleosome remodellers and cancer. Nat. Rev. Cancer 11, 481–492 10.1038/nrc3068 21654818

[B53] Winter J. Diederichs S. (2011). Argonaute proteins regulate microRNA stability. RNA Biol. 8, 1149–1157 10.4161/rna.8.6.17665 21941127

[B54] Yu J. Xiao J. Ren X. Lao K. Xie X. S. (2006). Probing gene expression in live cells, one protein molecule at a time. Science 311, 1600–1603 10.1126/science.1119623 16543458

[B55] Zhang Q. Bhattacharya S. Andersen M. E. (2013). Ultrasensitive response motifs: basic amplifiers in molecular signalling networks. Open Biol. 3:130031 10.1098/rsob.130031 23615029 PMC3718334

